# Role of Caryophyllane Sesquiterpenes in the Entourage Effect of Felina 32 Hemp Inflorescence Phytocomplex in Triple Negative MDA-MB-468 Breast Cancer Cells

**DOI:** 10.3390/molecules26216688

**Published:** 2021-11-05

**Authors:** Silvia Di Giacomo, Alessia Mariano, Marco Gullì, Caterina Fraschetti, Annabella Vitalone, Antonello Filippi, Luisa Mannina, Anna Scotto d’Abusco, Antonella Di Sotto

**Affiliations:** 1 Department of Physiology and Pharmacology “V. Erspamer”, Sapienza University of Rome, P.le Aldo Moro 5, 00185 Rome, Italy; marco.gulli@uniroma1.it (M.G.); annabella.vitalone@uniroma1.it (A.V.); 2 Department of Biochemical Sciences, Sapienza University of Rome, P.le Aldo Moro 5, 00185 Rome, Italy; alessia.mariano@uniroma1.it (A.M.); anna.scottodabusco@uniroma1.it (A.S.d.); 3 Department of Drug Chemistry and Technologies, Sapienza University of Rome, P.le Aldo Moro 5, 00185 Rome, Italy; caterina.fraschetti@uniroma1.it (C.F.); antonello.filippi@uniroma1.it (A.F.); luisa.mannina@uniroma1.it (L.M.)

**Keywords:** hemp inflorescences, phytocannabinoids, cannabidiol, cannabichromene, β-caryophyllene, β-caryophyllene oxide, α-humulene, chemosensitizing effects, triple negative breast cancer, entourage effect

## Abstract

*Cannabis sativa* L. crops have been traditionally exploited as sources of fibers, nutrients, and bioactive phytochemicals of medical interest. In the present study, two terpene-rich organic extracts, namely FOJ and FOS, obtained from Felina 32 hemp inflorescences collected in June and September, respectively, have been studied for their in vitro anticancer properties. Particularly, their cytotoxicity was evaluated in different cancer cell lines, and the possible entourage effect between nonintoxicating phytocannabinoids (cannabidiol and cannabichromene) and caryophyllane sesquiterpenes (β-caryophyllene, β-caryophyllene oxide and α-humulene), as identified at GC/MS analysis, was characterized. Modulation of cannabinoid CB1 and CB2 receptors was studied as a mechanistic hypothesis. Results highlighted marked cytotoxic effects of FOJ, FOS, and pure compounds in triple negative breast cancer MDA-MB-468 cells, likely mediated by a CB2 receptor activation. Cannabidiol was the main cytotoxic constituent, although low levels of caryophyllane sesquiterpenes and cannabichromene induced potentiating effects; the presence in the extracts of unknown antagonistic compounds has been highlighted too. These results suggest an interest in Felina 32 hemp inflorescences as a source of bioactive phytocomplexes with anticancer properties and strengthen the importance of considering the possible involvement of minor terpenes, such as caryophyllane sesquiterpenes, in the entourage effect of hemp-based extracts.

## 1. Introduction

*Cannabis sativa* L. (Fam. Cannabaceae) is a plant cultivated since the ancient times as a multipurpose crop: it is exploited all over the world to produce fiber, oil, and biomass that are used in different materials such as clothing, net, paper, canvas, varnishes, inks, biofuel, material for phytoremediation, food and animal feed, nutraceuticals, and cosmetics [1]. Recently, the interest in its medical uses is growing, owing to the highlighted therapeutic potential of its phytoconstituents, particularly Δ9-tetrahydrocannabinol (Δ9-THC or THC) and cannabidiol (CBD), which have been shown to possess numerous bioactivities, among which anticancer properties [2,3,4]. Based on the THC and CBD content, different crops of hemp have been approached for medical purposes; among them, type I Cannabis, characterized by high levels of THC (>85% *w*/*w*), and type II crops, mainly containing CBD, with relative high amounts of THC, have been limited due to the psychoactive effects of THC; by contrast, type III Cannabis, also known as industrial hemp, contains high levels of CBD (>85% *w*/*w*) and a very low amount of THC (less than 0.2% *w*/*w*).

Recently, due to the risk of intoxicating effects by THC [4], a great attention has been directed towards the therapeutical role of CBD and CBD-based extracts from industrial hemp [5,6,7]. Several studies have been focused on the ability of CBD to affect growth and progression of different cancer cells, such as glioma, neuroblastoma, and breast cancer, inducing both antiproliferative and antimetastasizing effects [4]. Likewise, anticancer properties have been highlighted for cannabichromene (CBC), a nonintoxicating phytocannabinoid usually found in low amounts in *C. sativa* varieties, in comparison to THC and CBD, except for some American Cannabis strains [4].

Despite the pharmacological interest in pure phytocannabinoids, *C. sativa* is known to possess a rich and variable phytocomplex, containing hundreds of other compounds, among which are monoterpenes and sesquiterpenes, which could contribute to the phytocomplex bioactivities; among these, the sesquiterpene β-caryophyllene is a common terpene in many cannabis varieties [1,4,6].

Some noteworthy studies have highlighted that the inflorescence extracts of cannabis were more active than the single purified phytocannabinoids [8,9,10,11]. For instance, Pagano et al. [12] found that crude CBD-rich cannabis extracts were more effective than pure CBD in reducing gastrointestinal inflammation in an in vivo model. Moreover, crude CBD-rich cannabis extract produced a linear anti-inflammatory and antinociceptive dose response in mice, despite the biphasic response of pure CBD [13]. Blasco-Benito et al. [10] have shown that a crude THC-based cannabis extract produced more potent antitumor effects than pure THC in breast cancer cell lines and animal models. Similarly, Namdar and colleagues [6] have found that the mixtures of THC or CBD with their co-related phytocannabinoids at naturally occurring ratios determined a greater cell cytotoxicity in comparison to the pure compounds alone.

The so called “entourage effect,” originated from synergistic interactions among cannabis phytochemicals, could explain this different behaviour, although the true mechanism involved must still be clarified [4,8]. It can arise from the interactions among different phytocannabinoids or among phytocannabinoids and other secondary metabolites, especially terpenes (e.g., monoterpenes and sesquiterpenes): these phenomena are defined as intra- and inter-entourage effects, respectively [6]. While the former has been deeply investigated [14], only few studies have explored the contribution of other compounds in the inter-entourage effect and the interactions among specific terpenes and the main phytocannabinoids [6,10].

Both intra- and inter-entourage effects can be affected by each hemp crop’s peculiar metabolomic composition, which arises from genetic features, cultivation conditions, and environmental factors, leading to different and variable bioactive responses with respect to pure compounds. Indeed, Berman et al. [15] highlighted that five different cannabis extracts, containing equal CBD amounts, produced different anticonvulsant effects, thus strengthening the importance of a comprehensive characterization of cannabis metabolome, including phytocannabinoids, terpenes, and minor metabolites, to obtain reproducible responses of hemp extracts and to support further pharmaceutical development of hemp-based extracts.

In line with this evidence, the present study was aimed at investigating the in vitro anticancer properties of terpene-based phytocomplexes from the inflorescences of *C. sativa* var. Felina 32 inflorescences. We focused our attention on Felina 32, which is an industrial hemp crop successfully exploited in textile and nutraceutical fields for stem and seeds [16]. Moreover, it is characterized by high yield in inflorescences, which can be reevaluated as possible by-products of pharmaceutical and medical interest.

To perform the study, Felina 32 inflorescences collected in June and September were subjected to Bligh-Dyer extraction, thus obtaining two terpene-rich organic fractions [17], here named FOJ (Felina 32 organic fraction from inflorescences collected in June) and FOS (Felina 32 organic fraction from inflorescences collected in September).

Cytotoxicity of the extracts was evaluated in different cancer cell lines, and the contribution of the major compounds detected at GC/MS, including the nonintoxicating phytocannabinoids (cannabidiol and cannabichromene) and caryophyllane sesquiterpenes (β-caryophyllene, β-caryophyllene oxide and α-humulene), was characterized. Possible entourage effects between phytocannabinoids and caryophyllane sesquiterpenes were assessed in combination studies. Being that the endocannabinoid system is a crucial target of phytocannabinoids [18], the ability of the extracts, pure compounds, and their combinations to modulate cannabinoid CB1 and CB2 receptors was studied as a mechanistic hypothesis.

## 2. Results

### 2.1. Absolute Quantitation of Nonintoxicating Phytocannabinoids and Caryophyllane Sesquiterpenes in FOJ and FOS Extracts from the Inflorescences of Cannabis sativa var. Felina 32

According to the GC-MS results, the content (expressed as percentage by weight) of nonintoxicating phytocannabinoids in FOJ and FOS was 8% and 34% for cannabidiol and 0.4% and 12% for cannabichromene, while that of caryophyllane sesquiterpenes was 0.09% and 0.15% for α-humulene, 0.09% and 0.19% for β-caryophyllene, and 0.07% and 0.21% for β-caryophyllene oxide. Cannabidiol was the most abundant nonintoxicating phytocannabinoid, with content about 20- and 3-times higher than cannabichromene in FOJ and FOS, respectively (Table 1). Moreover, the amount of both compounds increased with ripening, especially in the case of cannabichromene, for which a 29-fold increase was observed (Table 1). Regarding caryophyllane sesquiterpenes, equal amounts of α-humulene and β-caryophyllene were highlighted in the FOJ extract, despite a lower content of β-caryophyllene oxide. By contrast, FOS extract contained higher levels of β-caryophyllene and its epoxide analogue with respect to α-humulene. Comparing the extracts, the amount of caryophyllane sesquiterpenes was 2- to 3-folds higher in FOS than in FOJ.

### 2.2. Cytotoxicity of FOJ, FOS, Nonintoxicating Phytocannabinoids, and Caryophyllane Sesquiterpenes in Different Human Cancer Cell Lines

**Figure 1:**
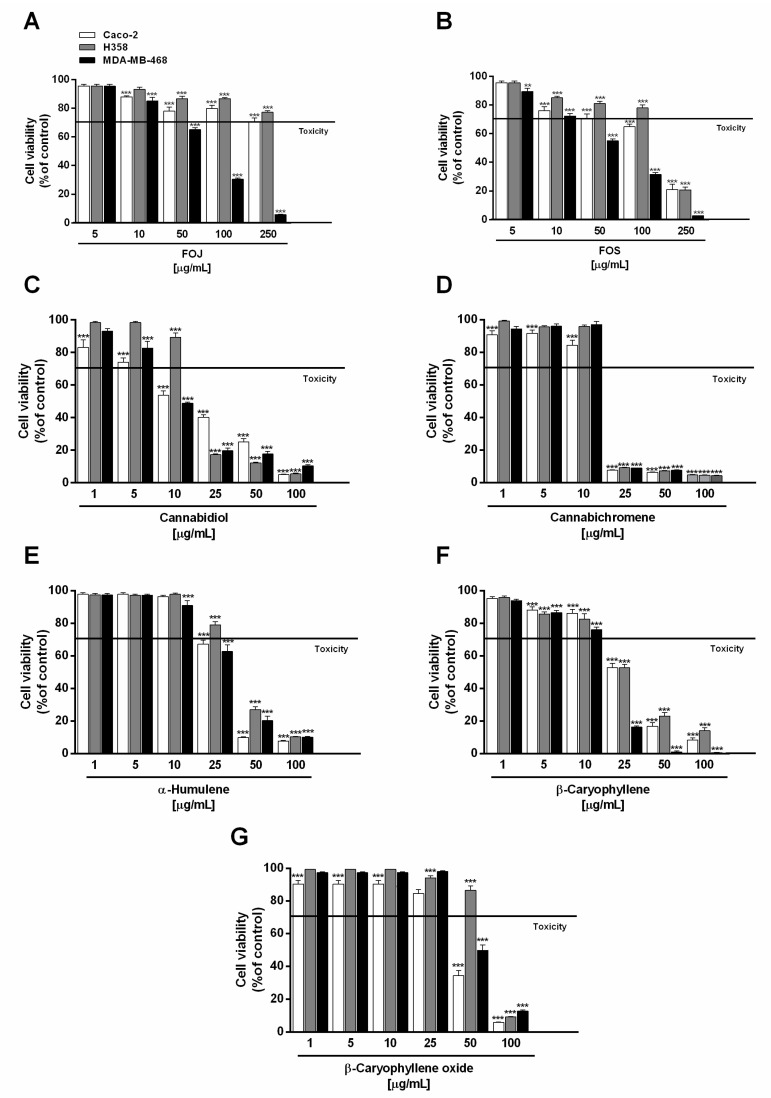
Cytotoxic effect of the organic extracts from the inflorescences of *Cannabis sativa* var. Felina 32 collected in June (FOJ) and September (FOS) (**A**,**B**), cannabidiol (**C**), cannabichromene (**D**), α-humulene (**E**), β-caryophyllene (**F**), and β-caryophyllene oxide (**G**) after 24 h treatment of human triple negative breast cancer (MDA-MB-468), human epithelial colorectal adenocarcinoma (Caco-2), and human bronchoalveolar carcinoma (H358) cells. Data are expressed as mean ± SEM of at least three independent experiments with three technical replicates (*n* = 9); ** *p* < 0.01 and *** *p* < 0.001 vs. control determined by ANOVA followed by Dunnett’s multiple comparison post hoc test.

Under our experimental conditions, the extracts (concentration range 5–250 µg/mL) significantly lowered MDA-MB-468 breast cancer cell viability, with a greater potency of FOS. A cell viability reduction by about 35% and 45% with respect to the control was induced by 50 µg/mL FOJ and FOS, respectively, achieving a higher than 95% inhibition at the maximum concentration tested (Figure 1A,B). According to the resulting IC_50_ values, FOJ potency was about 1.2-folds lower than that of FOS (Table 2).

H358 and Caco-2 cells were shown to be only slightly susceptible to the cytotoxic effects of FOJ, with cell viability higher than 70% even at the highest concentration tested (Figure 1A). Conversely, 250 µg/mL of FOS produced about an 80% reduction of H358 and Caco-2 cell viability, although it was poorly cytotoxic at the lower concentrations (Figure 1B). As evidenced by the IC_50_ values (Table 2), FOS stands out for its marked cytotoxicity in all the cell lines, with a greater potency in MDA-MB-468 breast cancer cells (IC_50_ approximately 1.4 and 2.0 times lower than those in Caco2 and H358).

In order to evaluate the possible bioactive compounds of FOJ and FOS, the cytotoxicity of the major nonintoxicating phytocannabinoids (i.e., cannabidiol and cannabichromene) and caryophyllane sesquiterpenes (i.e., α-humulene, β-caryophyllene, and β-caryophyllene oxide), as revealed at GC-MS analysis, was studied.

Under our experimental conditions, cannabidiol and cannabichromene were the most effective compounds in reducing cancer cell viability, followed by β-caryophyllene, especially in MDA-MB-468 cells. Cannabidiol produced about a 50% reduction of cell viability at the concentration of 10 µg/mL in both MDA-MB-468 and Caco-2 cells, being slightly effective against H358 cells (Figure 1C). Conversely, a marked cytotoxicity (about an 80% reduction of cell viability) was achieved at the concentrations of 25 µg/mL in MDA-MB-468 and H358 cells, and of 50 µg/mL in Caco-2 cells (Figure 1C).

Comparing the IC_50_ values, cannabidiol was about 1.7 and 1.3 times more potent in MDA-MB-468 than in Caco-2 and H358 cells, respectively (Table 2). Regarding cannabichromene, a maximum 90% inhibition of cell viability was found at the concentration of 25 µg/mL in all the cancer cell lines, with only slight cytotoxic effects at the lower concentrations (Figure 1D); accordingly, comparable IC_50_ values were determined (Table 2).

Among caryophyllane sesquiterpenes, α-humulene and β-caryophyllene produced significant cytotoxic effects in all the cancer cells starting from the concentration of 25 µg/mL, with a higher potency of the latter (Figure 1E,F). Indeed, it lowered cell viability of H358 and Caco-2 cells by about 45%, despite a 20 to 30% reduction induced by α-humulene. Interestingly, MDA-MB-468 cell viability was almost completely abolished (about an 80% lowering) by β-caryophyllene in comparison to a 40% inhibition by α-humulene. Conversely, the sesquiterpenes exhibited a similar behaviour starting from the 50 µg/mL concentration, reducing cell viability by at least 70% compared to the control. β-Caryophyllene oxide was the less potent cytotoxic sesquiterpene in all the cancer cells tested: at the concentration of 50 µg/mL, it produced about a 45% and 60% cell viability lowering in Caco-2 and MDA-MB-468 cells respectively, with only slight effects in H358 cells (Figure 1G); by contrast, a higher than 80% inhibition was achieved at 100 µg/mL.

Comparing the IC_50_ values, β-caryophyllene was approximately 1.8- and 3-times more potent than α-humulene and β-caryophyllene oxide in MDA-MB-468 cells, respectively; conversely, similar IC_50_ values were found for β-caryophyllene and α-humulene in Caco-2 and H358 cells (Table 2). β-Caryophyllene oxide was about 1.4- to 1.8-times less potent than β-caryophyllene and α-humulene in Caco-2 and H358 cells. The higher potency of β-caryophyllene in MDA-MB-468 cells suggests that the effect could be closely related to specific structural features, which remain to be defined.

Altogether, these results highlight that cytotoxicity of hemp extracts and pure compounds, especially cannabidiol and β-caryophyllene, is more selective towards MDA-MB-468 breast cancer cells; therefore, these cells have been selected to carry out further combination assays and mechanistic experiments.

### 2.3. Synergistic Activity among Nonintoxicating Phytocannabinoids and Caryophyllane Sesquiterpenes

To investigate the possible contribution of nonintoxicating phytocannabinoids and caryophyllane sesquiterpenes to FOJ and FOS cytotoxicity, MDA-MB-468 were treated for 24 h with a combination of them. Being that cannabidiol is the most cytotoxic and abundant compound, its combination with the other substances was evaluated; moreover, in order to highlight possible synergistic interactions, the pure compounds were assessed at the concentrations corresponding to those present in subtoxic concentrations of hemp extracts. To this end, 50 μg/mL of FOJ and FOS, which produced 30% to 40% inhibition of MDA-MB-468 viability, was selected.

Considering the GC-MS results, the following concentrations of nonintoxicating phytocannabinoids and caryophyllane sesquiterpenes, in 50 μg/mL of FOJ and FOS, respectively, were tested: 4 and 17 μg/mL for cannabidiol, 0.2 and 6 μg/mL for cannabichromene, 0.05 and 0.07 μg/mL for α-humulene, 0.05 and 0.1 μg/mL for β-caryophyllene, and 0.03 and 0.1 μg/mL for β-caryophyllene oxide.

**Figure 2:**
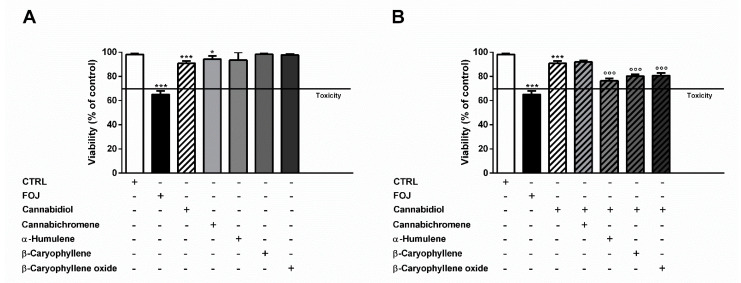
(**A**) Cytotoxicity of 50 μg/mL FOJ (organic extract from the inflorescences of *Cannabis sativa* var. Felina 32 collected in June), and of nonintoxicating phytocannabinoids (4 μg/mL cannabidiol, 0.2 μg/mL cannabichromene) and caryophyllane sesquiterpenes (0.05 μg/mL α-humulene, 0.05 μg/mL β-caryophyllene, and 0.03 μg/mL β-caryophyllene oxide), at the corresponding concentrations determined in the extract by GC-MS analysis, in MDA-MB-468 after 24 h exposure. (**B**) Cytotoxicity of 4 μg/mL cannabidiol alone and in combination with cannabichromene (0.2 μg/mL), α-humulene (0.05 μg/mL), β-caryophyllene (0.05 μg/mL), and β-caryophyllene oxide (0.03 μg/mL) in MDA-MB-468 after 24 h exposure. Data are expressed as mean ± SEM of at least three independent experiments with three technical replicates (*n* = 9); * *p* < 0.05 and *** *p* < 0.001, significant difference respect to the control; °°° *p* < 0.001, significant difference respect to cannabidiol (ANOVA + Dunnett’s Multiple Comparison PostTest).

As displayed in Figure 2A, 4 μg/mL cannabidiol reduced the cell viability by about 10%, despite a 35% cytotoxicity of FOJ, thus suggesting that the hemp extract activity cannot be ascribed to the only cannabidiol. The other compounds did not affect cell viability at the concentrations found in FOJ, except for a slight reduction induced by cannabichromene (Figure 2A). Despite lacking cytotoxic effects, α-humulene (0.05 μg/mL), β-caryophyllene (0.05 μg/mL), and β-caryophyllene oxide (0.03 μg/mL) induced a slight but significant 1.1- to 1.2-times potentiation of cannabidiol (4 μg/mL) cytotoxicity; by contrast, cannabichromene (0.2 μg/mL) did not show any synergistic effect (Figure 2B).

**Figure 3:**
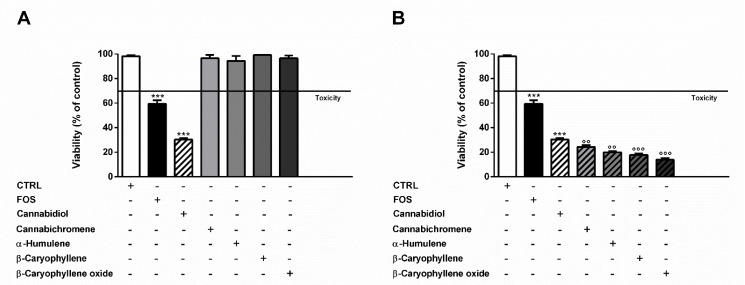
(**A**) Cytotoxicity of 50 μg/mL FOS (organic extract from the inflorescences of *Cannabis sativa* var. Felina 32 collected in September), and of nonintoxicating phytocannabinoids (17 μg/mL cannabidiol, 6 μg/mL cannabichromene) and caryophyllane sesquiterpenes (0.07 μg/mL α-humulene, 0.1 μg/mL β-caryophyllene, and 0.1 μg/mL β-caryophyllene oxide), at the concentrations determined in the extract by GC-MS analysis, in MDA-MB-468 after 24 h exposure. (**B**) Cytotoxicity of 17 μg/mL cannabidiol alone and in combination with cannabichromene (6 μg/mL), α-humulene (0.07 μg/mL), β-caryophyllene (0.1 μg/mL), and β-caryophyllene oxide (0.1 μg/mL) in MDA-MB-468 after 24 h exposure. Data are expressed as mean ± SEM of at least three independent experiments with three technical replicates (*n* = 9); *** *p* < 0.001, significant difference respect to the control; °° *p* < 0.01 and °°° *p* < 0.001, significant difference respect to cannabidiol (ANOVA + Dunnett’s Multiple Comparison PostTest).

Otherwise, at the concentration of 17 μg/mL measured in FOS, cannabidiol produced a 2-times higher cytotoxic effect than the extract, despite null effects of the other compounds (Figure 3A). Combining α-humulene (0.07 μg/mL), β-caryophyllene (0.1 μg/mL), β-caryophyllene oxide (0.1 μg/mL), and 17 μg/mL cannabidiol, an increased cytotoxicity by about 1.5-, 1.7-, and 2.2-times was achieved, respectively. Furthermore, cannabichromene (6 μg/mL) produced a slight 1.2-fold increase of cannabidiol cytotoxicity (Figure 3B).

**Figure 4:**
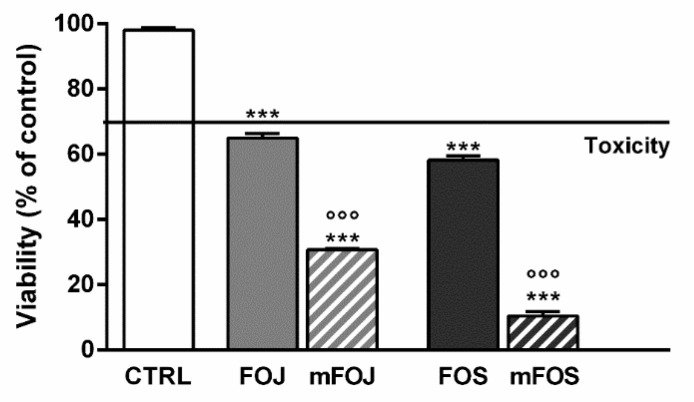
Cytotoxicity of 50 μg/mL FOJ and FOS (organic extracts from Felina 32 hemp inflorescences collected in June and September respectively) and the corresponding cocktails mFOJ and mFOS (containing nonintoxicating phytocannabinoids and caryophyllane sesquiterpenes at the same concentrations found in 50 μg/mL FOJ and FOS) in MDA-MB-468 breast cancer cells. Data are expressed as mean ± SEM of at least three independent experiments with three technical replicates (*n* = 9); *** *p* < 0.001, significant difference respect to the control; °°° *p* < 0.001, significant difference respect to Felina 32 organic extracts (ANOVA + Dunnett’s Multiple Comparison PostTest).

In order to confirm the involvement of nonintoxicating phytocannabinoids and caryophyllane sesquiterpenes in the cytotoxicity of the extracts, we also tested the effects of two cocktails, namely mFOJ and mFOS, prepared by mixing cannabidiol, cannabichromene, α-humulene, β-caryophyllene, and β-caryophyllene oxide to achieve the same final concentrations determined in 50 μg/mL FOJ and FOS. Intriguingly, mFOJ and mFOS produced cytotoxic effects higher than those of the hemp extracts, being 2.1- and 5.6-times more effective than FOJ and FOS, respectively (Figure 4). This confirms the involvement of the tested mixtures in the cytotoxic effects of the extracts and suggests the presence of other unknown compounds, likely responsible for antagonistic interactions.

### 2.4. FOJ and FOS Induced Cytotoxicity by Targeting the Endocannabinoid System

**Figure 5:**
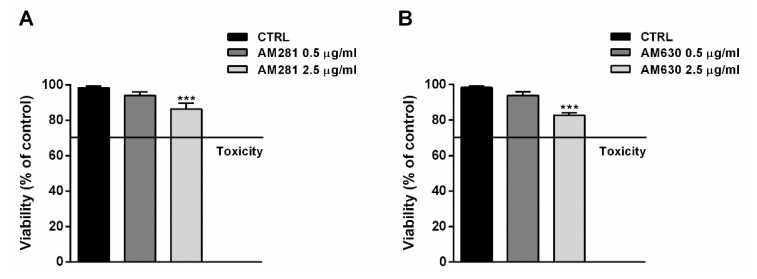
Effect of AM281 (**A**) and AM630 (**B**) antagonists on the viability of MDA-MB-468 breast cancer cells. The cells were treated with antagonists for 1 h, then washed and further incubated for 24 h. Data are expressed as mean ± SEM of at least three independent experiments with three technical replicates (*n* = 9); *** *p* < 0.001, significant difference respect to the control (ANOVA + Dunnett’s Multiple Comparison PostTest).

The possible involvement of the endocannabinoid system in the cytotoxicity of FOJ and FOS (50 µg/mL) was evaluated in MDA-MB-468 by treating cells for 1 h with the specific CB1 and CB2 receptor antagonists AM281 and AM630 before a 24 h exposure to the extracts. The antagonist concentrations, i.e., 0.5 and 2.5 µg/mL, were chosen on the basis of literature [19], and their lack of cytotoxicity was confirmed in preliminary experiments (Figure 5A,B).

**Figure 6:**
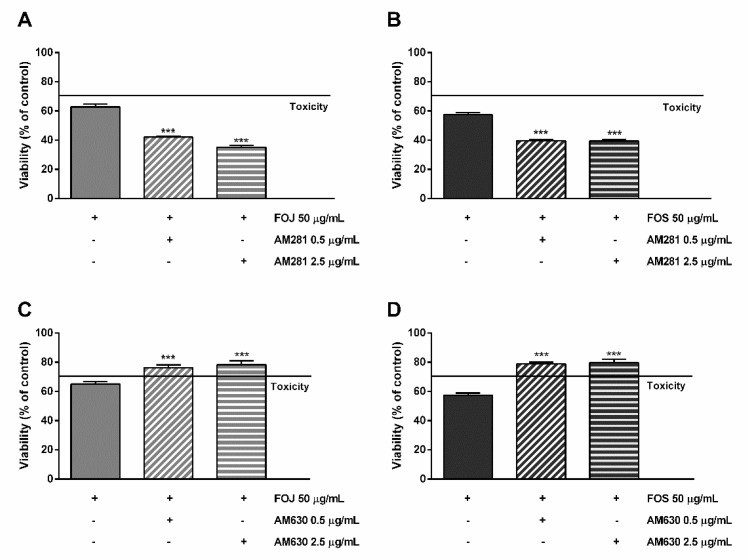
Cytotoxicity of 50 μg/mL FOJ and FOS (organic extracts from Felina 32 hemp inflorescences collected in June and September respectively) after treatment with 0.5 and 2.5 µg/mL AM281 (**A**,**B**) and AM630 (**C**,**D**) in MDA-MB-468 breast cancer cells. The cells were subjected to a 1 h pre-treatment with the antagonist, then incubated with the tested samples for 24 h. Data are expressed as mean ± SEM of at least three independent experiments with three technical replicates (*n* = 9); *** *p* < 0.001, significant difference respect to the control (ANOVA + Dunnett’s Multiple Comparison PostTest).

As displayed in Figure 6A,B, the cytotoxicity of FOJ and FOS was not diminished by AM281. Indeed, increasing the inhibitor concentration, a progressive 1.5- to 1.8-fold lowering in cell viability with respect to FOJ was induced; similarly, FOS cytotoxicity increased in the presence of AM281 by about 1.4- and 1.5-times. Conversely, both the AM630 concentrations significantly hindered FOJ and FOS cytotoxicity, with a reduction by about 1.2- and 1.4-times, respectively (Figure 5C,D).

**Figure 7:**
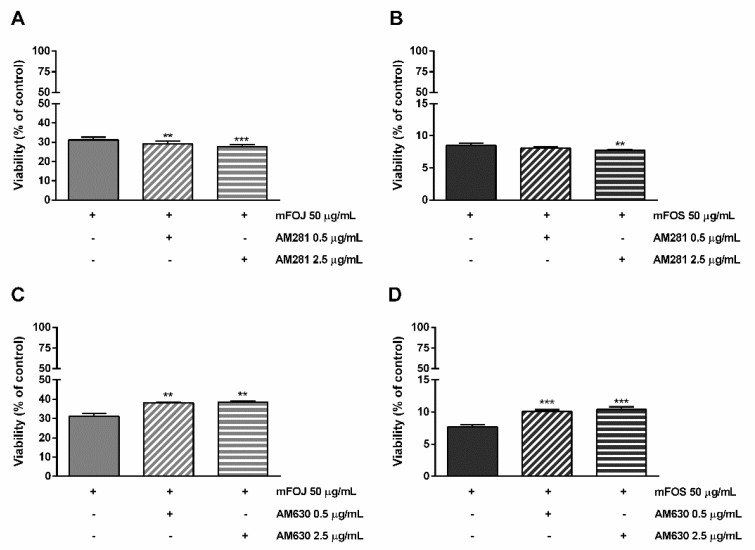
Cytotoxicity of the mFOJ and mFOS cocktails (containing the same concentrations of nonintoxicating phytocannabinoids and caryophyllane sesquiterpenes determined in 50 μg/mL FOJ and FOS) after treatment with 0.5 and 2.5 µg/mL AM281 (**A**,**B**) and AM630 (**C**,**D**), in MDA-MB-468 breast cancer cells. The cells were subjected to a 1 h pre-treatment with the antagonist, then incubated with the tested samples for 24 h. Data are expressed as mean ± SEM of at least three independent experiments with three technical replicates (*n* = 9); ** *p* < 0.01 and *** *p* < 0.001, significant difference respect to the control (ANOVA + Dunnett’s Multiple Comparison PostTest).

Likewise, the pre-treatment of MDA-MB-468 cells with AM281 produced a slight but significant increase of the mFOJ and mFOS cocktail cytotoxicity (Figure 6A,B); on the other hand, AM630 significantly reduced the effects of mFOJ and mFOS by about 1.2- and 1.3-folds, respectively (Figure 7C,D).

Present results suggest the involvement of both CB1 and CB2 receptors in the control of breast cancer cell proliferation; moreover, the cytotoxicity of FOJ and FOS extracts and of mFOJ and mFOS cocktails seems to be not ascribable to a CB1 receptor modulation, despite an involvement, albeit partial, of CB2 receptors.

### 2.5. Modulation of CB2 Receptor Expression

In order to confirm the hypothesis of the involvement of a CB2 receptor modulation in the cytotoxicity of FOJ and FOS extracts and mFOJ and mFOS cocktails, and to verify the presence of these receptors in the tested cells, immunofluorescence analysis in the presence of a suitable anti-CB2 antibody has been performed. To this end, the nontoxic concentration of 10 μg/mL was chosen for both FOJ and FOS; the corresponding mFOJ and mFOS cocktails were prepared and tested too. Moreover, the possible modulation of CB2 receptor expression by nonintoxicating phytocannabinoids and caryophyllane sesquiterpenes (i.e., 0.8 and 3.4 μg/mL cannabidiol; 0.04 and 1.2 μg/mL cannabichromene; 0.01 and 0.02 μg/mL α-humulene; 0.01 and 0.02 μg/mL β-caryophyllene; 0.007 and 0.02 μg/mL β-caryophyllene oxide, contained in 10 μg/mL FOJ and FOS) was assessed.

**Figure 8:**
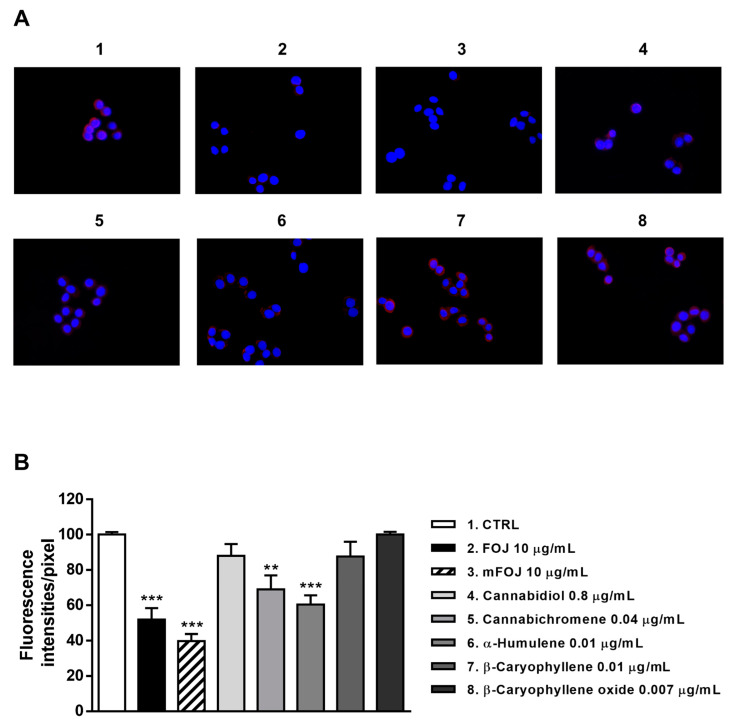
Modulation of CB2 receptor expression in MDA-MB-468 cells detected after 24 h treatment with 10 μg/mL FOJ (organic extract from Felina 32 hemp inflorescences collected in June), the corresponding mFOJ cocktail, and with the nonintoxicating phytocannabinoids and caryophyllane sesquiterpenes contained in FOJ extract. (**A**) Representative images obtained at immunofluorescence analysis. (**B**) Densitometric analysis of immunofluorescence carried out by the free software ImageJ (*n* = 6). ** *p* < 0.01 and *** *p* < 0.001, significant difference respect to the control (ANOVA + Dunnett’s Multiple Comparison PostTest). 1. Control; 2. FOJ; 3. mFOJ; 4. Cannabidiol, 0.8 μg/mL; 5. Cannabichromene, 0.04 μg/mL; 6. α-Humulene, 0.01 μg/mL; 7. β-Caryophyllene, 0.01 μg/mL; 8. β-Caryophyllene oxide, 0.007 μg/mL.

**Figure 9:**
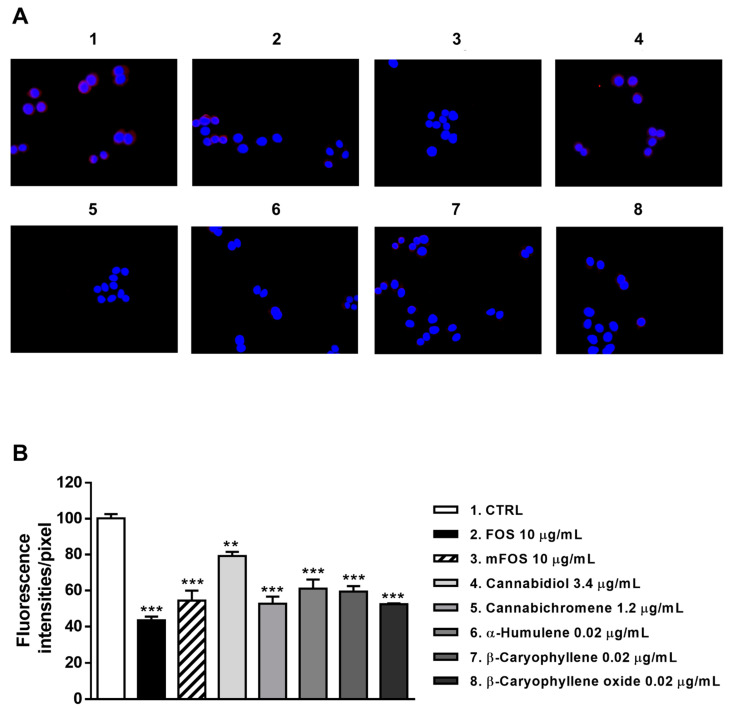
Modulation of CB2 receptor expression in MDA-MB-468 cells detected after 24 h treatment with 10 μg/mL FOS (organic extract from Felina 32 hemp inflorescences collected in September), the corresponding mFOS cocktail, and with the nonintoxicating phytocannabinoids and caryophyllane sesquiterpenes contained in FOS extract. (**A**) Representative images obtained at immunofluorescence analysis. (**B**) Densitometric analysis of immunofluorescence carried out by the free software ImageJ (*n* = 6). ** *p* < 0.01 and *** *p* < 0.001, significant difference respect to the control (ANOVA + Dunnett’s Multiple Comparison PostTest). 1. Control; 2. FOS; 3. mFOS; 4. Cannabidiol, 3.4 μg/mL; 5. Cannabichromene, 1.2 μg/mL; 6. α-Humulene, 0.02 μg/mL; 7. β-Caryophyllene, 0.02 μg/mL; 8. β-Caryophyllene oxide, 0.02 μg/mL.

Staining of MDA-MB-468 cells with a specific antibody highlighted the presence of CB2 receptors on their surface, as shown by a red color in control cells, which appeared differently when modulated by the treatments (Figure 8A and Figure 9A). After 24 h treatment, FOJ and mFOJ were able to significantly decrease CB2 receptor expression by about 1.9- and 2.5-times, respectively, despite a lower modulation by 0.04 μg/mL cannabichromene (1.4-fold reduction) and 0.01 μg/mL α-humulene (1.6-fold reduction). Conversely, 0.8 μg/mL cannabidiol, 0.01 μg/mL β-caryophyllene, and 0.007 μg/mL β-caryophyllene oxide did not affect CB2 receptor expression (Figure 8A,B).

Similarly, CB2 receptor expression in MDA-MB-468 was significantly affected by FOS and mFOS, with a 2.3- and 1.8-fold decrease, respectively. Among nonintoxicating phytocannabinoids, 1.2 μg/mL cannabichromene lowered the receptor expression 1.9-times, with a slight 1.2-times reduction induced by 3.4 μg/mL cannabidiol (Figure 9A,B). All the caryophyllane sesquiterpenes (0.02 μg/mL α-humulene, β-caryophyllene, and β-caryophyllene oxide) affected CB2 receptor expression, inducing a 1.6- to 1.8-fold reduction (Figure 9A,B). These results suggest that the lowered presence of CB2 receptors could be a consequence of their previous activation, thus supporting our hypothesis about the involvement of a CB2 receptor modulation in the cytotoxicity of Felina 32 hemp extracts and its major terpenes.

## 3. Discussion

In line with the growing pharmacological interest in industrial hemp as the source of bioactive compounds and phytocomplex [2,3,6,10,13], in the present study, the in vitro anticancer properties of the organic FOJ and FOS extracts from Felina 32 inflorescences, collected in June and September, respectively, and the possible entourage effect among their major nonintoxicating phytocannabinoids (cannabidiol and cannabichromene), and caryophyllane sesquiterpenes (β-caryophyllene, β-caryophyllene oxide and α-humulene), have been investigated.

As revealed at GC-MS analysis, FOJ and FOS were characterized by a growing content of cannabidiol, cannabichromene, β-caryophyllene, β-caryophyllene oxide and α-humulene over the seasons. This trend partly agrees with previous evidence obtained by Aizpurua-Olaizola et al. [20], studying different fiber-type hemp plants grown indoors under controlled conditions during the flowering period. Similarly to our samples, a progressive increase of cannabidiol content despite a low amount of cannabichromene, but no changes in the levels of caryophyllane sesquiterpene, was found [20]. These differences could be due to genetic and ontogenetic features of plants, cultivation conditions, and environmental factors, and confirm the peculiar composition of each hemp phytocomplex and its possible impact on the bioactivity profile.

Under our experimental conditions, both FOJ and FOS extracts significantly reduced the cell viability of tested cell lines, although with more selectivity towards MDA-MB-468 breast cancer cells and a higher potency of FOS. Similarly, cancer cell viability was significantly lowered by pure compounds, with cannabidiol, cannabichromene, and β-caryophyllene being the most effective, especially in MDA-MB-468 cells.

A great attention has been devoted over the years to the possible usefulness of cannabis and hemp extracts for cancer treatment, with the role of phytocannabinoids THC and CBD [10,21] being especially highlighted, with a lower contribution of polyphenols [22]. Particularly, Blasco-Benito et al. [10] highlighted a similar or slightly greater potency of a THC-rich cannabis extract with respect to pure THC in reducing the viability of different breast cancer cells, although minor identified terpenes did not affect THC effects. Furthermore, several studies have reported the anticancer properties of cannabidiol in both in vitro and in vivo preclinical models [4,21,23,24]; recently some evidence about the anticancer activity of cannabichromene, β-caryophyllene, β-caryophyllene oxide, and α-humulene have been highlighted as well [4,25,26].

Despite the results achieved by Blasco-Benito et al. [10], we found a lower anticancer potency of FOJ and FOS extracts in comparison to the pure compounds, especially CBD: this could be due to the presence of compounds likely acting in an antagonistic way in the extracts. Moreover, previous evidence has highlighted that the dose ratio between the phytocannabinoids plays a crucial role in determining the kind of interaction [27]. Indeed, about a 1.8 ratio between CBD/THC produced potentiating effects, whereas a much higher ratio of about 8.1 leads to antagonistic effects [27].

Considering the amount of nonintoxicating phytocannabinoids and caryophyllane sesquiterpenes detected at GC-MS analysis, the IC_50_ of FOJ in MDA-MB-468 cells corresponds to 7.5 μg/mL cannabidiol, which is about 1.3-fold lower than the IC_50_ of pure CBD. Therefore, the FOJ cytotoxicity seems to be not ascribable to the only cannabidiol, but the contribution of other compounds appears likely. Conversely, the amount of cannabidiol at IC_50_ of FOS in MDA-MB-468 cells is 26.8 μg/mL, which is almost 3-fold higher than the IC_50_ of pure CBD. After assessing the cytotoxic effects of the corresponding mixtures mFOJ and mFOS in MDA-MB-468 cells, a 2.1- and 5.6-times higher effectiveness than FOJ and FOS was found, respectively. This supports our hypothesis about the presence of antagonistic compounds towards CBD in our extracts, albeit different from cannabichromene, β-caryophyllene, β-caryophyllene oxide, and α-humulene.

In order to better disclose the role of other compounds in the activity of hemp extracts, we performed combination studies in MDA-MB-468 breast cancer cells, which resulted in the most sensitive model under our experimental conditions. Interestingly, caryophyllane sesquiterpenes were found able to increase cannabidiol cytotoxicity at the nontoxic and very low concentrations found in FOJ and FOS extracts; conversely, cannabichromene produced only a slight cannabidiol potentiation. Although previous studies highlighted a weak correlation of β-caryophyllene and THCA (Δ9-tetrahydrocannabinolic acid) and CBDA (cannabidiolic acid) from which THC and CBD arise [6], our results showed a chemosensitizing role of caryophyllane sesquiterpenes towards cannabidiol in FOJ and FOS, which can contribute to the inter-entourage effect. These apparently contradictory findings can be due to the differences in the tested extracts and in the specific ratio between phytocannabinoids and minor terpenes. The authors [6] point out the need of more specifically studying the combinations of phytocannabinoids and terpenes and their effective ratios for achieving the inter-entourage effect; for instance, significantly higher ratios than those produced by plant have been found not effective.

Under our experimental conditions, the ratio between CBD and cannabichromene was 19 and 2.8 in FOJ and FOS respectively, although an intra-entourage effect was found only in the second combination, which was also similar to the optimal ratio found for CBD/THC [27]. Regarding caryophyllane sesquiterpenes, ratios of 85 and 227, 87 and 179, and 118 and 162 were found in FOJ and FOS for cannabidiol and β-caryophyllene, cannabidiol and β-caryophyllene oxide, and cannabidiol and α-humulene, respectively. All of them produced potentiating effects of cannabidiol, thus suggesting a possible contribution in the inter-entourage effect in FOJ and FOS.

Previous evidence has highlighted the ability of caryophyllane sesquiterpenes to synergize the effects of different anticancer drugs likely acting as chemosensitizing agents [25]. Particularly, β-caryophyllene and β-caryophyllene oxide displayed chemosensitizing properties in combination with low-dose doxorubicin and sorafenib, likely by a modulation of ABC-transporters, mainly P-glycoprotein (Pgp) and multidrug resistance-associated proteins 1 (MRP1) and 2 (MRP2), but at a lower extent of BCRP (unpublished data) [28,29,30]. Multiple inhibitory mechanisms, including a direct interaction in the transporter binding site, a modulation of protein expression, and a possible interference with the pump conformation due to an alteration in the membrane permeability [31], have been hypothesized [28,29,30]. In this regard, a recent study has shown that phytocannabinoids are substrates of ABC transporters [32], therefore, the pump inhibition mediated by caryophyllane sesquiterpenes could effectively determine an increase of phytocannabinoids into the cells and then an increased cytotoxicity.

β-Caryophyllene, β -caryophyllene oxide, and α-humulene, have been also found to induce apoptotic cancer cell death through the regulation of different pathways, such as JAK1/STAT3, NF-kB and PI3K/AKT/mTOR/S6K1 [4,25,33,34]. Particularly, STAT3 protein is involved in cancer progression, metastasization, and drug resistance, thus, its inhibition can represent an interesting strategy for increasing cancer chemotherapy sensitivity [33]. Moreover, in some cancer models, apoptosis induced by β-caryophyllene has been ascribed to a modulation of CB2 receptors [4,25].

Overall, these mechanisms can contribute to the chemosensitizing properties of caryophyllane sesquiterpenes, thus leading to an increased cannabidiol cytotoxicity: this can partly explain the mechanisms accounting for the inter-entourage effects in the tested extracts.

To deeply investigate the mechanism by which FOJ and FOS extracts and the mixture of nonintoxicating phytocannabinoids and caryophyllane sesquiterpenes induced the antiproliferative effects in the triple negative breast cancer cell model, the involvement of the endocannabinoid system, particularly the possible modulation of CB1 and CB2 receptors, was assessed. Indeed, the activation of CB receptors determines an antitumorigenic effect by inhibiting tumor cell proliferation, inducing apoptosis, and blocking angiogenesis and tumor metastasis [35]. Moreover, many preclinical studies and histological tumor samples have shown that more aggressive tumors present an upregulation of these receptors; particularly, triple-negative breast cancers, the most aggressive form often associated with a poor prognosis, is known to highly express CB2 receptors [36]. Therefore, targeting CB-associated pathways could be a promising treatment option.

Our results highlighted that blocking CB1 receptors contributes to the inhibition of breast cancer cell growth, although these receptors were not targeted by our samples. Conversely, the modulation of CB2 receptors has highlighted as a mechanism involved in the cytotoxicity of both FOJ and FOS extracts and the mixtures mFOJ and mFOS.

This evidence has been corroborated by immunofluorescence analysis, which highlighted a decreased expression of CB2 receptors on the cell surface, especially due to the treatment with the extracts, FOJ and FOS mixtures and caryophyllane sesquiterpenes. This reduction can be a consequence of receptor activation; indeed, following the agonist binding, CB2 receptors are subjected to internalization, which in turn determines the activation of other signaling pathways inaccessible to receptors residing on the surface membrane [37]. This phenomenon strengthens our hypothesis about the involvement of CB2 receptor activation in the cytotoxicity of our samples.

The involvement of CB2 receptors on the chemosensitizing effects of caryophyllane sesquiterpenes towards cannabidiol agree with previous published studies. Indeed, β-caryophyllene has been highlighted to selectively activate the cannabinoid CB2 receptors [38], thus leading to apoptosis death of cancer cells [39]. Moreover, it modulates further targets in the endocannabinoidome, such as the peroxisome proliferator-activated receptors (PPARs) and the fatty acid amide hydrolase (FAAH) [25,40]. Similarly, β-caryophyllene oxide has been reported to be a CB2 receptor agonist in an in vivo study [41]. Conversely, a direct interaction between α-humulene and the CB2 receptor has not been proven yet. Altogether, these results support our hypothesize about the modulation of CB2 receptors by caryophyllane sesquiterpenes and stimulate further studies to better understand this outlined mechanism of action.

Regarding cannabidiol, it is known to act as a multitarget agent, being an inverse agonist of CB2 receptor, and an antagonist of the non-cannabinoid GPR55 receptor, the transient receptor potential cation channel subfamily M member 8 (TRPM8), and the T-type Ca^2+^ channels; moreover, it has been shown to inhibit the fatty acid amide hydrolase (FAAH), responsible for the degradation of anandamide, and the fatty acid-binding protein (FABP), which favors the uptake of anandamide into cell, making it available for intracellular targets, such FAAH or nuclear PPARγ [35,42]. These mechanisms lead to an increased extracellular anandamide concentration, which can indirectly activate CB1 and CB2 receptors [35,42].

Overall, present results highlight Felina32 hemp inflorescences to be a source of bioactive phytocomplexes, containing an interesting combination between nonintoxicating phytocannabinoids and caryophyllane sesquiterpenes to be exploited in cancer research and strengthen the importance of considering minor terpenes of the hemp metabolome, such as caryophyllane sesquiterpenes, due to their possible involvement in the inter-entourage effects. Moreover, a modulation of diverse endocannabinoid targets and molecular signallings by the tested extracts seems to be likely: further studies could allow to better characterize the mechanisms accounting for the in vitro anticancer properties of Felina 32 hemp extracts and to confirm their efficacy in vivo. However, the optimal ratio between nonintoxicating phytocannabinoids and caryophyllane sesquiterpenes to maximize the anticancer properties of Felina 32 hemp extracts remains to be disclosed.

## 4. Materials and Methods

### 4.1. Chemical and Reagents

The chemicals β-caryophyllene (≥98.5% purity), β-caryophyllene oxide (≥99% purity), α-humulene (≥96.0% purity), cannabidiol (CBD, ≥99.0% purity), cannabichromene (CBC, ≥99.0% purity), 3-[4,5-dimethylthiazol-2-yl]-2,5-diphenyl tetrazolium bromide (MTT, ≥97.5% purity) were purchased from Sigma Aldrich Co (St. Louis, MO, USA), while AM281 (≥98.0% purity) and AM630 (≥98.0% purity) from Tocris bioscience (Bristol, UK). Dulbecco’s Modified Eagle’s Medium (DMEM) was provided by Aurogene (Rome, Italy).

To perform the experiments, all the solutions were prepared in the appropriate solvent, sterilized, and stored at the recommended temperature for a conservation time. Nonintoxicating phytocannabinoids (i.e., cannabidiol and cannabichromene) and caryophyllane sesquiterpenes (i.e., β-caryophyllene, β-caryophyllene oxide, and α-humulene) were dissolved in EtOH 100% *v*/*v*, while the CB1 and CB2 receptor antagonists (i.e., AM281 and AM630) in DMSO 100% *v*/*v*. EtOH and DMSO were used at a maximum concentration of 1% *v*/*v* in cell medium to avoid any cytotoxicity.

### 4.2. Organic Extracts from Felina 32 Hemp Inflorescences

The tested terpene-rich organic fractions, namely FOJ (Felina 32 Organic fraction from June) and FOS (Felina 32 Organic fraction from September), were obtained by the Bligh-Dyer extraction of Felina 32 hemp inflorescences, as previously reported [16].

Plant material was supplied by “Canapa Live” cultural association and harvested at the cultivation site in Santa Severa Nord (Lazio region, Central Italy) at both early and late flowering stages, namely June and September. Particularly, 30 plants in the central part of the cultivation area were collected for inflorescence sampling, and the upper part (30 cm) of the stem was cut; inflorescences were then merged to obtain a unique representative sample of each harvesting time and stored at −80 °C [17].

Phytochemical analysis of FOJ and FOS highlighted that the extracts contained different terpenes and polyphenols, which grew over the seasons. Among them, the nonintoxicating phytocannabinoids CBD and cannabichromene as well as the caryophyllane sesquiterpenes α-humulene, β-caryophyllene, and β-caryophyllene oxide were the most abundant identified compounds; conversely, THC level was always under the limit required by the Italian law (max 0.2% *w*/*w*) for industrial hemp [17].

### 4.3. Absolute Quantitation of Nonintoxicating Phytocannabinoids and Caryophyllane Sesquiterpenes

Absolute quantitation of nonintoxicating phytocannabinoids and caryophyllane sesquiterpenes was made by Gas Chromatography/Mass Spectrometry (GC/MS). To this end, FOJ and FOS were dissolved in ethanol to a final 1 mg/mL concentration and then analyzed using an Agilent Technologies 6850 gas chromatograph coupled with an Agilent Technologies 5975 mass spectrometer, equipped with HP-5MS capillary column (5% Phenyl 95% methylpolysiloxane, 30 m × 0.25 mm i.d., film thickness 0.25 µm; Hewlett-Packard, CA, USA).

GC parameters were adjusted as follows: injector temperature of 250 °C, flow rate of the helium carrier gas (99.995% purity) 1.0 mL/min. The oven temperature was kept at 40 °C for 5 min, then raised to 200 °C (5 °C/min) and maintained at this temperature for 60 min. MS parameters were set as follows: energy of electron ionization 70 eV, solvent delay 6 min, source temperature 230 °C, quadrupole temperature 150 °C, and mass scan carried out over the 50–350 *m*/*z* range. The analysis was performed in triplicate, with two replicates for each experiment.

The resulting chromatogram presented two prevalent classes of compounds, namely sesquiterpenes and phytocannabinoids. Three sesquiterpenes (α-humulene, β-caryophyllene and its derivative β-caryophyllene oxide) and two nonintoxicating phytocannabinoids (cannabidiol and cannabichromene) have been selected as the most representative compounds of both classes and therefore quantified through the internal standard method (IS = 4-phenyl-2-butanol).

A calibrated solution containing IS and weighted amounts of the selected compounds have been analyzed for calculating the response factor of each analyte. Therefore, 1 mg/mL solution of FOJ and FOS in presence of 10^−2^ mg/mL IS have been analyzed under the same chromatographic conditions.

### 4.4. Cancer Cell Cytotoxicity

#### 4.4.1. Cell Culture

Human MDA-MB-468 triple negative breast cancer cells were provided by Interlab Cell Line Collection (IRCCS San Martino Policlinico Hospital, Genova, Italy), while Caco2 epithelial colorectal adenocarcinoma cells were obtained from American Type Culture Collection (ATCC). H358 bronchoalveolar carcinoma cells were a kind gift of Prof. Fabio Altieri (Department of Biochemical Sciences “Alessandro Rossi Fanelli”, Rome, Italy). The cells were grown under standard conditions (37 °C and 5% CO_2_) in Dulbecco’s Modified Eagle Medium (DMEM) medium, containing L-glutamine (1% *v*/*v*), HEPES (15 mM), 100 U/mL penicillin, 100 μg/mL streptomycin, and 10% heat inactivated FBS in 75 cm^2^ flasks. Cells were subcultured every 4 days, renewing growth medium twice a week, as recommended by the supplier.

#### 4.4.2. Cytotoxicity Assay

Confluent cells were seeded into 96-well microplates (2 × 10^4^ cells/well), allowed to grow for 24 h, then treated with progressive concentrations (5, 10, 50, 100 and 250 μg/mL) of FOJ and FOS for 24 h. The same exposure protocol was used to evaluate the cytotoxicity of the major identified phytocannabinoids, i.e., cannabidiol and cannabichromene (1 to 100 μg/mL concentration range, corresponding to 3–318 μM), caryophyllane sesquiterpenes, i.e., β-caryophyllene, β-caryophyllene oxide, α-humulene (1 to 100 μg/mL concentration range corresponding to 5–500 μM), and the positive control doxorubicin. At the end of incubation, cytotoxicity of the treatments was determined by the MTT assay, according to previous methods [43], and absorbance was measured by using a microplate reader (Epoch Microplate Spectrophotometer, BioTeK Instruments Inc., Winooski, VT, USA). To obtain reproducible data, at least three biologic replicates, in which each concentration was tested in triplicate, were made. Comparing the number of viable cells in each treatment with respect to the vehicle control allowed measurement of a cell viability reduction: a treatment was considered cytotoxic when the cell viability was less than 70% with respect to vehicle [33].

### 4.5. Combination Assay

To study the possible synergistic interactions between nonintoxicating phytocannabinoids and caryophyllane sesquiterpenes in the cytotoxicity of hemp organic extracts, combination experiments (co-treatment protocol of 24 h exposure to the tested substances) in MDA-MB-468 cells were made, according to Di Sotto et al. [33]. Particularly, the cytotoxicity of cannabidiol was evaluated in combination with cannabichromene, β-caryophyllene, β-caryophyllene oxide, and α-humulene at the concentrations measured in FOJ and FOS was tested.

Moreover, two cocktails of the nonintoxicating phytocannabinoids and caryophyllane sesquiterpenes, namely mFOJ and mFOS respectively, containing the same concentrations determined in the FOJ and FOS extracts, were prepared. In the same experiments, suitable controls with the substances alone were tested too. At the end of the 24 h exposure, cell viability was measured by MTT assay, as previously reported.

### 4.6. Modulation of Endocannabinoid System by Felina 32 Extracts and Pure Compounds

To evaluate the possible involvement of the endocannabinoid systems in the cytotoxicity of the tested samples, the cells were exposed to FOJ and FOS extracts (50 µg/mL) and to the cocktails mFOJ and mFOS in the presence of AM281 and AM630, which are selective antagonists of CB1 and CB2 receptors, respectively.

Based on literature [19] and preliminary experiments, the nontoxic concentrations of 0.5 and 2.5 µg/mL, corresponding to 1 and 5 µM, were chosen to be tested for both AM630 and AM281. According to the experimental protocol, the antagonists were administered one hour before treatment with the test samples; after a 24 h exposure, cell viability was measured by the MTT assay.

### 4.7. Immunofluorescence Analyses

CB2 receptors were visualized by immunofluorescence, as previously reported [44]. Cells were plated at a density of 3 × 10^3^/cm^2^ and cultured for 24 h, washed in PBS, then fixed in methanol for 2 min and permeabilized with 0.5% Triton-X 100 in PBS for 10 min at room temperature. After blocking with 3% bovine serum albumin (BSA) in PBS for 30 min at room temperature, cells were incubated for 1 h, at room temperature, with mouse monoclonal anti-CB2 antibody 1:150 (Santa Cruz Biotechnology, Inc., Dallas, TE, USA). Cells were washed with PBS and then incubated for 1 h, at room temperature, with Alexa Fluor 594 donkey anti-mouse antibody 1:400 (Invitrogen, Thermo Fisher Scientific, Waltham, MA, USA) to stain receptors in red. Slides were washed and then stained with DAPI (Invitrogen, Thermo Fisher Scientific) to visualize the nuclei. The images were captured by a Leica DM IL LED optical microscope, using an AF6000 modular microscope (Leica Microsystem, Milan, Italy). The free software ImageJ (https://imagej.nih.gov/ij/) (accessed on 24 August 2021) was used to perform the densitometric analysis.

### 4.8. Statistical Analysis

All values are expressed as mean ± SE of at least two or three independent experiments with three technical replicates (*n* = 6 or *n* = 9). Statistical analysis was performed by GraphPad Prism™ (Version 5.00) software (GraphPad Software, Inc., San Diego, California, USA). Differences among treatments were evaluated by the one-way analysis of variance (one-way ANOVA), followed by Dunnett’s multiple comparison post-test. The concentration–response curves were obtained by “Hill equation”: E = E_max_/[1 + (10^LogEC^_50_/A)^HillSlope^], where E is the effect at a given concentration of agonist, E_max_ is the maximum activity, EC_50_ is the concentration that produces a 50% of the inhibitory response (namely IC_50_), A is the agonist concentration, HillSlope is the slope of the agonist curve. *p* values < 0.05 and <0.01 were considered as significant, and very significant, respectively.

## 5. Conclusions

In this study, the entourage effects accounting for the in vitro anticancer activity of the FOJ and FOS terpene-rich extracts, obtained from the inflorescences of Felina 32 industrial hemp, have been investigated. We highlighted a contribution of low-level caryophyllane sesquiterpenes to the inter-entourage effect of both extracts, along with an intra-entourage of a 3:1 combination of cannabidiol and cannabichromene. Antagonistic interactions, arising from unknown compounds in the extracts, have been displayed too.

Moreover, the ratio among bioactive compounds appears crucial to achieve optimal entourage effects, albeit difficult to clearly define, due to the enormous variability in the studied extracts and the richness of hemp phytochemicals to be considered. In this intricate scenario, the unique combinations designed by nature in crops can be approached as a starting point to clarify the question and to select the suitable conditions to be exploited for further pharmacological interest.

Altogether, these findings strengthen the importance to deeply characterize the entire hemp metabolome, including phytocannabinoids, minor terpenes, and polyphenols, and their role as possible active (or co-active) pharmaceutical ingredients, with improvements on standardization and therapeutic efficacy of hemp formulations.

## Figures and Tables

**Table 1:** Absolute concentration of nonintoxicating phytocannabinoids and caryophyllane sesquiterpenes in FOJ and FOS extracts from the inflorescences of *Cannabis sativa* var. Felina 32 collected in June and September, respectively. Data are expressed as µg ± SE per mg of extract (*n* = 6).

Compound	FOJ	FOS
	µg ± SE in 1 mg Extract	
Cannabidiol	80.00 ± 7.40	340.00 ± 27.00
Cannabichromene	4.20 ± 0.70	120.00 ± 3.70
α-Humulene	0.94 ± 0.05	1.50 ± 0.20
β-Caryophyllene	0.92 ± 0.04	1.90 ± 0.20
β-Caryophyllene oxide	0.68 ± 0.01	2.10 ± 0.10

**Table 2:** Half maximal inhibitory concentration (IC_50_) values of the organic extracts from the inflorescences of *Cannabis sativa* var. Felina 32 collected in June (FOJ) and September (FOS), nonintoxicating phytocannabinoids, caryophyllane sesquiterpenes, and the positive control doxorubicin in human MDA-MB-468 triple negative breast cancer, Caco-2 epithelial colorectal adenocarcinoma and H358 broncho alveolar carcinoma cells after 24 h exposure.

Sample	MDA-MB-468	Caco-2	H358
	IC_50_ (CL) ^a^ µg/mL		
FOJ	96.9 (81.6–115.2)	- ^b^	- ^b^
FOS	78.9 (69.8–92.8)	111.8 (51.9–240.7)	158.2 (81.61–272.9)
Cannabidiol	9.3 (6.9–12.4)	16.2 (8.6–30.6)	12.1 (11.2–13.0)
Cannabichromene	15.7 (2.9–85.1)	13.2 (2.6–65.2)	15.9 (2.8–89.1)
α-Humulene	28.8 (23.9–34.8)	27.9 (25.9–29.9)	35.2 (33.3–37.2)
β-Caryophyllene	15.9 (4.5–57.2)	31.9 (25.1–39.2)	32.3 (25.1–41.5)
β-Caryophyllene oxide	49.3 (46.4–52.3)	44.4 (41.3–47.8)	59.4 (53.9–65.3)
Positive control ^c^	1.6 (1.4–1.7)	8.4 (3.2–9.8)	13.5 (8.1–22.5)

^a^ CL, confidence limits; ^b^ -, not evaluable being a lower than 80% inhibition achieved. ^c^ Doxorubicin.

## Data Availability

The data are included in the main text.

## Sample Availability

The samples of compounds “cannabidiol, cannabichromene, β-caryophyllene, β-caryophyllene oxide and α-humulene” are available from authors.
